# The Bidirectional Relationship Between Obstructive Sleep Apnea and Metabolic Disease

**DOI:** 10.3389/fendo.2018.00440

**Published:** 2018-08-06

**Authors:** Sarah N. Framnes, Deanna M. Arble

**Affiliations:** Department of Biological Sciences, Marquette University, Milwaukee, WI, United States

**Keywords:** sleep apnea, leptin, glucose, diabetes, obesity, insulin, metabolism, disordered breathing

## Abstract

Obstructive sleep apnea (OSA) is a common sleep disorder, effecting 17% of the total population and 40–70% of the obese population (1, 2). Multiple studies have identified OSA as a critical risk factor for the development of obesity, diabetes, and cardiovascular diseases (3–5). Moreover, emerging evidence indicates that metabolic disorders can exacerbate OSA, creating a bidirectional relationship between OSA and metabolic physiology. In this review, we explore the relationship between glycemic control, insulin, and leptin as both contributing factors and products of OSA. We conclude that while insulin and leptin action may contribute to the development of OSA, further research is required to determine the mechanistic actions and relative contributions independent of body weight. In addition to increasing our understanding of the etiology, further research into the physiological mechanisms underlying OSA can lead to the development of improved treatment options for individuals with OSA.

## Obstructive sleep apnea: clinical presentation and preclinical models

Obstructive sleep apnea (OSA) is a common sleep disorder classically characterized by apneic events leading to intermittent hypoxia and sleep fragmentation. OSA is most commonly found in obese, middle age men (6). Obesity is strongly associated with OSA (7) with approximately 40–70% of the obese population diagnosed with OSA (1, 2). Unfortunately, OSA has broad detrimental effects on health ranging from increased daytime sleepiness to a 4-fold increase in mortality (1). As the name implies, OSA derives from obstruction of the airway. While the cause of obstruction varies between individuals, common obstructions occur due to abnormal anatomy [e.g., narrow airway, enlarged tonsils (8)], obese anatomy [e.g., increased fat storage in pharyngeal tissue (9, 10)], and/or decreased neuromuscular tone (11). During a polysomnography evaluation in the sleep laboratory, an individual with OSA experiences periods of breathing reduction (hypopnea) or cessation (apnea) coincident with respiratory effort. The severity of an individuals' apnea and hypopnea is defined by the apnea-hypopnea index (AHI). An individual with mild OSA experiences 5–15 apnea-hypopnea events per hour, whereas those with moderate or severe OSA experience 15–30 or >30 events/h, respectively (12). Apneic events lead to reductions in blood oxygen saturation, and over the course of the night, present as intermittent hypoxia (IH) (13). It is estimated that an individual with severe OSA may reach blood oxygen saturation levels as low as ~76% (14) and it is widely regarded that these drops in oxygen play a key role in many of the downstream disease states associated with OSA. Reduction in blood oxygen and elevations in blood carbon dioxide are sensed by chemoreceptors in the brain and carotid bodies, which trigger brief microarousals and result in sleep fragmentation (15). These repeated microarousals are believed to contribute to Excessive Daytime Sleepiness (EDS), another characteristic of OSA. EDS, as scored by the Epworth Sleepiness Scale, measures an individuals' perceived sleepiness. Higher levels of EDS are associated with an increased risk of falling asleep at work or driving, and is associated with decreased life satisfaction (15). In a large sleep study, 76% of individuals with severe OSA exhibited EDS, and 56% of individuals with mild or moderate OSA exhibited EDS (16). In addition to apneic events, an individual with OSA exhibits a blunted hypercapnic ventilatory response (HCVR) and a blunted hypoxic ventilatory response (HVR) (17), demonstrating impaired chemosensitivity. Interestingly, blunted HCVR (18) and HVR (19, 20) are observed in some obese patients without OSA, most often those with obesity hypoventilation syndrome, suggesting that impaired chemosensitivity may occur before the onset of apneic events.

In contrast to OSA, central sleep apnea (CSA) is defined by the cessation of air flow *without* perceived respiratory effort (21). Like OSA, individuals with CSA may exhibit multiple apneas throughout the night. While CSA effects <5% of individuals referred to the sleep clinic (22), an increased risk for CSA is observed in individuals with compromised chemoreception. For example, CSA is found in ~24% of chronic opioid users (23) due to opioid-induced impairments to the carotid bodies and hypoglossal nerve signaling (23). Interestingly, ~13–20% of individuals diagnosed with OSA exhibit central apneas as well (24, 25). In particular, individuals with type 2 diabetes (T2D) have an increased chance of experiencing both OSA and CSA (i.e., mixed apnea) (26). Increased recognition of mixed apneic events has led to an emerging hypothesis which postulates that OSA and CSA share common mechanisms of action (22).

Currently, continuous positive airflow pressure, or CPAP, is the most effective and widely used treatment for OSA (27). By delivering a continuous flow of air, CPAP actively keeps the airway open and can improve the AHI of OSA patients an average of ~13 events/h (28). Despite the dramatic improvement in AHI from CPAP treatment, compliance is low. Only 39–50% of users will use CPAP (29) for the recommended minimum of at least 4 h per night for 5 days per week (30). Thus, improved treatment strategies for OSA are needed.

Yet, despite the prevalence of OSA, the serious health risks, and the inadequate treatment options, we have a poor understanding of how sleep apnea develops. While clinical studies have been instrumental in laying the foundation of OSA research, basic science approaches using rodent models have enabled investigators to explore the etiology of OSA. Initially, the English bulldog was used as a naturally occurring model of OSA which exhibited snoring, sleep disordered breathing, and daytime sleepiness (31). While it was first believed that the apnea of the English bulldog was occurring solely due to abnormalities of the upper airway (e.g., narrow nares and enlarged soft palate), these anatomical features only accounted for a subset of apneic events. Indeed, during sleep studies, English bulldogs displayed apneic events *without* respiratory effort, representative of central sleep apnea (31). While the English bulldog was a good initial model, and mirrored humans by exhibiting naturally occurring OSA (23), it also experienced apnea in a lean state. To better account for the obesity observed in many OSA individuals, lean and obese Yucatan miniature pigs were utilized as another naturally occurring model of OSA (32). Similar to the English bulldog, obese pigs experienced mixed apneic events, however, lean pigs did not experience any apneic events (32). These data suggested that obesity may be a key factor contributing to sleep apnea. While Yucatan miniature pigs were a naturally occurring model of OSA, further mechanistic studies were difficult owing to sheer size of the animals and lack of available genetic tools.

Currently, much of the mechanistic hypotheses involving OSA are tested in rodent models. The rodent offers superior capabilities in behavioral and genetic manipulation, allowing more detailed investigation into the mechanisms leading to the metabolic consequences of OSA. To examine sleep apnea in rodent models, researchers have modeled two main characteristics of OSA: sleep fragmentation and intermittent hypoxia (IH). In general, sleep loss and decreased sleep quality without the presence of OSA is associated with obesity, impairments in glucose regulation, and reductions in insulin sensitivity (33). While IH is associated with many of these same outcomes, IH often leads to weight loss instead of weight gain, perhaps due to the observed increases in circulating leptin (see leptin section below). Since IH mirrors both the oxygen desaturation as well as the microarousals associated with OSA (15), many of the mechanistic hypotheses on OSA and metabolism have sprouted from IH studies. A wealth of data indicates that chronic IH results in profound impairments in cardiometabolism similar to those experienced by individuals with OSA, including hypertension (34), ventricular hypertrophy (35), insulin resistance, and hyperlipidemia (36, 37). Using whole-body plethysmography, researchers have also observed similarities between the chemosensitivity of obese rodents (measured via the ventilatory responses to hypercapnia and hypoxia, with and without IH) to that of individuals with OSA (38, 39). In this way, the ventilatory responses of rodents presents itself as another measure analogous to the physiology of individuals with OSA. Diet-induced obese rodents can also be used alongside lean controls to determine the effect of obesity on ventilation parameters and IH-induced outcomes. Indeed, just as in humans (18), diet-induced obesity leads to a depressed ventilatory response in rodent models (38, 40). Using a combination of clinical and rodent studies, investigators can significantly increase our understanding of the etiology of sleep apnea.

## The etiology of OSA and its bidirectional relationship with metabolic disease

In some individuals, OSA etiology is clearly associated with anatomical obstruction. For example, most OSA diagnosed in children is due to enlarged tonsils and is treated with tonsillectomy (41). However, many clinical evaluations for OSA do not reveal any obvious anatomical obstructions (42, 43). In the absence of a clear anatomical obstruction, much of the etiological theory on OSA has focused on one of its most profoundly associated factors: obesity.

Multiple studies have shown a clear, positive association between obesity and AHI (7). More specifically, increased visceral obesity (44) and neck circumference (45) have been linked to OSA. While it is generally accepted that obesity is an important prerequisite for OSA, the hypothesized mechanisms by which obesity contributes to OSA vary widely. Indeed, the relative contributions of an individuals' physical weight vs. an individuals' metabolic physiology in the development of OSA is an active area of debate (46–48).

**Figure 1:**
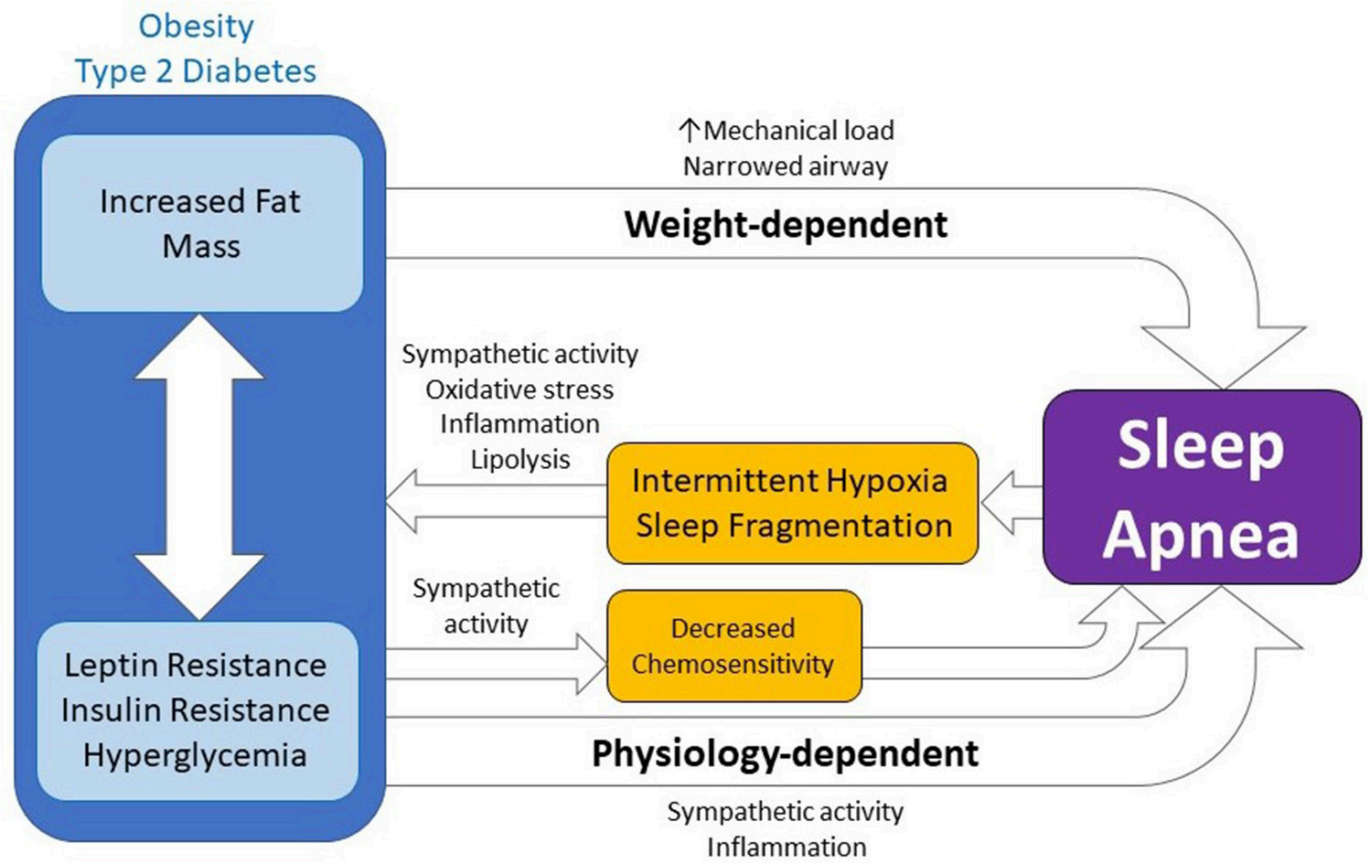
The bidirectional relationship between obstructive sleep apnea and metabolic disease. Sleep apnea results in intermittent hypoxia and sleep fragmentation which lead to and exacerbate obesity and type 2 diabetes by increasing sympathetic activity, oxidative stress, inflammation, and lipolysis. Moreover, metabolic disease can lead to, or exacerbate, sleep apnea through weight-dependent and physiology-dependent mechanisms. While weight-dependent mechanisms are a function of the physical increase in body mass or fat mass (e.g. increased mechanical load, narrowed airway), physiology-dependent mechanisms are physiological changes coincident with obesity or diabetes which go on to influence chemosensitivity and sleep apnea either directly or via action on sympathetic activity, inflammation, or other mechanisms.

Traditionally, the strong association between obesity and OSA led many to conclude that OSA occurred due to increased fat mass mechanically restricting airflow. Specifically, increased fat deposits in the tongue and/or larger pharyngeal tissue (9, 10, 49) were hypothesized to be too heavy for the reduced muscular tone normally experienced during rapid-eye movement (REM) sleep and thus, the tissue's increased physical weight lead to an obstruction the airway and apnea or hypopnea. Increased physical mass also affects lung mechanics, reducing functional residual capacity and tidal volume (50). For the remainder of this review, we refer to mechanisms supporting this hypothesis as *weight-dependent* (Figure 1). However, focusing only on physical body weight as an underlying mechanism to OSA does not explain why only a subset of obese individuals have sleep apnea (51). Nor does physical body weight alone explain why lean individuals develop sleep apnea (52). Nevertheless, the association between obesity and OSA suggests that these variables may be related to each other in ways that go beyond the physical mechanical weight of fat.

There are two alternative explanations to the strong association between obesity and OSA. The first of which is that OSA is leading to obesity and metabolic dysfunction (Table 1). Indeed, OSA-associated IH and sleep fragmentation have been repeatedly found to induce and exacerbate cardiometabolic disease (91). This directional hypothesis is generally accepted and well-reviewed [see (53, 77, 92)]. Therefore, we only highlight key studies supporting this hypothesis in this review. Instead, we focus on a second intriguing possibility, that obese physiology and not physical weight *per se* leads to the development of OSA (Table 2).

**Table 1:** Summary of presented evidence that obstructive sleep apnea and its components are associated with decreased glycemic control, insulin resistance, increased leptin, and decreased chemosensitivity.

**Model**	**Results**	**References**
Obstructive sleep apnea (human)	↓ Hypoxic ventilatory response ↓ Hypercapnic ventilatory response ↓ Glycemic control ↑ Insulin resistance ↑ Leptin	(17, 52–66)
Type 2 diabetes + Obstructive sleep apnea	↓ Glycemic control ↑ Apnea-hypopnea index ↑ Central sleep apnea ↑ Insulin resistance	(26, 54–57, 67–75)
Sleep fragmentation	↓ Glycemic control ↑ Insulin resistance ↑ Leptin	(33, 76)
Intermittent hypoxia	↓ Glycemic control ↑ Insulin resistance ↑ Leptin ↓ Chemosensitivity	(36, 37, 77–89)
Obesity + Intermittent hypoxia	↑ Insulin resistance ↓ Hypoxic ventilatory response ↓ Hypercapnic ventilatory response	(39, 77, 90)

**Table 2:** Summary of presented evidence that the manipulation of glycemic control, insulin, and leptin are associated with increased apneic events and decreased chemosensitivity.

**Model**	**Results**	**References**
Metabolic surgery	↓ Apnea-hypopnea index	(93, 94)
Type 2 diabetes (poor glycemic control, insulin resistance)	↑ Apnea-hypopnea index ↑ Central sleep apnea	(26, 53, 71, 74, 75, 95)
Streptozotocin-treatment (destroys pancreatic β-cells)	↓ Apnea-hypopnea index ↓ Hypoxic ventilatory response ↓ Hypercapnic ventilatory response	(96, 97)
Type 1 diabetes (insulin deficient)	↑ Apnea-hypopnea index ↑ Central sleep apnea	(98, 99)
Polycystic ovary syndrome (insulin resistance)	↑ Apnea-hypopnea index	(100–102)
Metformin treatment (insulin sensitizer)	↓ Apnea-hypopnea index ↑ Chemosensitivity	(96, 97, 103)
Leptin impairment (leptin and/or leptin receptor deficiency)	↓ Hypoxic ventilatory response ↓ Hypercapnic ventilatory response	(104–108)
Lipodystophy (low leptin, insulin resistance)	↑ Apnea-hypopnea index	(109–113)

Emerging hypotheses postulate that physiological components of obesity, including glycemic control, insulin action, and leptin signaling, contribute to the development of OSA. It's possible that obese physiology leads to greater reductions in pharyngeal dilator muscle tone and results in increased chance of obstruction during sleep (114). This greater reduction in muscle tone may be due to chronically increased muscle activity, due to increased autonomic response, and/or histological changes to the muscle tissue itself via inflammatory pathways (49). Alternatively, obese physiology may be leading to disordered breathing and increased central sleep apnea via decreased chemosensitivity (18–20). Given the increased risk of mixed apneic events observed within type 2 diabetics, this latter observation is particularly interesting. Research on this front is on-going and it's possible that other mechanisms by which obese physiology impacts sleep apnea may soon be defined. Collectively, we refer to mechanisms that support these hypotheses as *physiology-dependent* or *weight-independent* (Figure 1).

Teasing apart the relative contribution of physical, weight-dependent mechanisms from physiological-dependent mechanisms is inherently difficult due to the close relationship between obesity and its associated changes in glycemic control, insulin action, and leptin signaling. For example, obesity is strongly associated with glucose dysregulation and weight loss alone can substantially improve fasting glucose and glucose tolerance within individuals with T2D (115). In the context of OSA, weight loss through dieting can also substantially improve AHI (116). However, it is unclear if these dramatic improvements in apneic symptoms are from weight-dependent or physiological-dependent mechanisms, as dieting both reduces physical body weight and improves glucose metabolism.

A unique way to partition the effect of weight loss from substantial changes in metabolic physiology has utilized data from bariatric surgical procedures. Bariatric surgical procedures, such as the Roux-en-Y Gastric Bypass (RYGB), the vertical sleeve gastrectomy (VSG), and the laparoscopic adjustable gastric band (LAGB) lead to significant, sustained weight loss and improvements in glucose regulation (117). However, RYGB and VSG are unique among bariatric surgical procedures in that glucose metabolism is improved through both weight-dependent and weight-independent mechanisms. In fact, due to their ability to improve glucose regulation in part through weight-independent mechanisms, RYGB and VSG are sometimes referred to as metabolic surgeries (118). This contrasts with the metabolic improvements following LAGB which parallel total weight loss without additional improvements from weight-independent means (117, 118). Following metabolic surgeries, improvements in glucose tolerance can occur quickly, before significant weight loss occurs (117, 118). In some cases, individuals can discontinue their diabetic medication before being discharged from the hospital (117). To determine how OSA may be affected by metabolic improvements independent of weight loss, it would be ideal to quantify OSA on a time scale before significant weight loss occurs. However, most polysomnography following bariatric surgical procedures occurs 6 months to 1 year post-operatively and thus after significant weight loss is achieved. However, quantifying EDS, closely related to OSA, can be done without polysomnography. In one study, individuals undergoing RYGB showed resolution of EDS symptoms within 1 month, accompanied by only marginal weight loss (119). While it is tempting to speculate that sleep apnea too may be improved on a time scale indicative of weight-independent mechanisms, this question remains unanswered. While EDS is associated with OSA, there is also an independent relationship between obesity and sleep. Overweight individuals are more likely to exhibit increased sleepiness during the day independent of OSA (120, 121). Moreover, decreased sleep duration and sleep quality has been linked to increases in BMI and metabolic dysfunction (122). Therefore, improvements in EDS following bariatric surgery could be a result of small to moderate changes in body weight and/or improvements in metabolic physiology independent of sleep apnea. Alternatively, directly comparing OSA outcomes following metabolic surgeries such as RYGB and VSG vs. weight-loss surgeries such as LAGB can provide insight into the relative contributions of weight-dependent and physiology-dependent mechanisms in the etiology of OSA. A number of comparative studies have reported that OSA resolution 1-year after RYGB or VSG is approximately double that of individuals undergoing LAGB (93, 94). Furthermore, other studies have shown that LAGB has no better OSA resolution compared to diet-induced weight loss, despite more weight loss attained via LAGB (123). Given the added weight-independent metabolic benefits following RYGB and VSG, these data suggest that some component of obese physiology and not body weight itself, may be involved in the etiology of OSA.

To better address how obese physiology may impact disordered breathing, investigators have incorporated preclinical animal models. Indeed, the preclinical setting allows researchers to systemically manipulate glycemic control, insulin sensitivity, and/or leptin and examine their specific contributions to disordered breathing. While we address each of these variables in detail in the sections below, a commonality among these experiments is the use of high-fat diets to induce obesity within the animal models. Similar to humans, diet-induced obesity leads to a depressed hypercapnic ventilatory response (40) and a restrictive ventilatory pattern (39) in mice. Importantly, since diet-induced obesity alone leads to both increased physical weight and metabolic syndrome, a more detailed approach (such as including weight as a covariate or using weight-matched controls) must be used to specifically determine how obese physiology contributes to disordered breathing. Moreover, the addition of high-fat diets has also been found to exacerbate the metabolic consequences of IH. Obese, high-fat fed mice exposed to chronic IH demonstrate further detriments in insulin resistance (39, 90), suggesting that obesity itself or obese physiology may exacerbate OSA disease outcomes.

## Glycemic control

A prominent characteristic of obese physiology is an impairment in glycemic control. Clinical association studies and randomized control trials have evaluated the relationship between OSA and glycemic control with mixed results. In support of an association between apnea and glycemic control, a recent pilot study found that the combination of respiratory events and nocturnal awakenings could predict variability of fasting blood glucose in T2D patients (67). Nocturnal hypoxemia has also been independently associated with the development of impaired glycemic control (54) and T2D in healthy individuals (55) and worsened glycemic control in individuals with T2D (68). Moreover, with the use of continuous glucose monitoring, T2D individuals with OSA have been shown to exhibit peaks in circulating glucose levels temporally following blood oxygen desaturation (69). Taken together, these studies demonstrate that OSA, and in particular nocturnal hypoxemia, likely leads to elevated glucose levels. In non-diabetic individuals, daily, 24-h rhythms in circulating glucose variability have been associated with OSA severity (56), suggesting that the association between OSA and improper glucose control may precede T2D. Whether circulating glucose levels directly impact disordered breathing or OSA is less clear. It would be informative to explore if individuals with recurrent hypoglycemia or nocturnal hypoglycemia are at increased risk for OSA and/or have reduced chemosensitivity (124). While unexplored, this information could advance our understanding of the involvement of glycemic control and/or glucose sensing in the development of sleep apnea.

In animal models, simulation of OSA using chronic IH has greatly advanced our knowledge of how OSA may impact disease states via cyclic drops in blood oxygen. Rodents exposed to chronic IH have increased gluconeogenesis in the liver (78–80), fasting hyperglycemia, and decreased glucose tolerance (81). Acute, 3-h, exposures of IH in healthy humans also leads to an increase in circulating glucose levels before noticeable changes to insulin sensitivity (125). Indeed, much of the effects on glycemic control from OSA may be attributed to IH (126). Moreover, altering metabolic state prior to IH impacts the outcome, indicating a bidirectional relationship between glycemic control and IH. For example, fasting can mitigate some cardiovascular consequences of IH, including the activation of glycogen synthase in the myocardium (127). Additionally, treatment with a lipolysis inhibitor ameliorates hyperglycemia and glucose intolerance induced by IH in mice (81), highlighting an important role for the adipose tissue and lipolysis in many of the downstream consequences of IH and perhaps OSA (77, 128). Taken together, it is likely that circulating and fasting glucose is increased by OSA and that elevated glucose before the theoretical onset of OSA is likely to exacerbate the cardiometabolic outcomes of OSA.

Another way to explore the relationship between glycemic control and OSA is by intervention and treatment studies. One would hypothesize that if alterations in glucose were downstream of OSA, then treatment of OSA alone would improve glycemic control. While there are randomized controlled studies which support this hypothesis (129), others report no improvement in glycemic control with CPAP use (130). One possibility for these conflicting results is the presence of existing glycemic impairment. For example, in a recent study, higher glycemic variability was associated with sleep disordered breathing in both T2D and non-diabetic individuals, however CPAP treatment only improved glycemic variability in those *without* T2D (57). Similarly, a meta-analysis concludes that CPAP may prevent the development of T2D in non-diabetic individuals (131), again pointing to the effectiveness of CPAP on glycemic control before T2D develops. However, withdraw from CPAP in both obese T2D and non-diabetics leads to an increase in nocturnal glucose without affecting glucose tolerance, production, or insulin (132), suggesting that CPAP use is leading to a reduction in glucose. Together, these data point to the likelihood that glucose impairment is downstream of OSA in non-diabetic individuals, but it remains to be elucidated the relationship between glycemic control and OSA within those with T2D.

One possible mechanism linking glucose dysregulation and OSA is via autonomic dysfunction. T2D leads to autonomic dysfunction and this directly affects respiratory control and cardiac outcomes consistent with the presentation of OSA (133). This mechanism is supported by impaired autonomic activity observed in individuals with central hypoventilation syndrome which exhibit sleep disordered breathing, hypoglycemia and hyperinsulinemia (134). Sympathetic activity is also directly involved in modulating fasting hyperglycemia following exposure to IH (135), pointing to the ability of the sympathetic system to modulate glucose metabolism in addition to respiratory outcomes (133). Chronic IH has also been observed to increase tonic and reactive afferent chemoreceptor outputs from the carotid body which in turn effects catecholamine to modulate the autonomic nervous system (82–84) and leads to fasting hyperglycemia (136) and hypertension (84). An interesting area of research positions the carotid bodies as key integrators of glucose metabolism, OSA, and autonomic function. Glomus cells in the carotid bodies sense oxygen, carbon dioxide, and glucose. Interestingly, oxygen and glucose signals can potentiate one another, leading to scenarios where dysregulation of glucose may lead to a dysregulation of O_2_ and CO_2_ sensing which in turn may affect breathing (137). Addition of 2-deoxy-d-glucose (2DG; a glucoprivic agent) in the drinking water of rats can prevent phrenic long-term facilitation, a form of respiratory motor plasticity, suggesting that alterations in glucose sensing can directly alter breathing (138). Work in this field is ongoing and shows great potential in elucidating bidirectional pathways between glucose control and disordered breathing via the sympathetic system.

## Insulin

A key player in glucose metabolism and tightly linked to obesity, insulin action has also been investigated in the context of OSA (95, 139). OSA is correlated with an increased risk of T2D (53) and within the diagnosed OSA population, approximately 15–30% exhibit symptoms of T2D (140, 141). Moreover, a meta-analysis of longitudinal studies concludes that the relative risk ratio of an individual with moderate/severe OSA developing T2D is 1.63 (95% CI: 1.09–2.45) compared to an individual without significant apneic events (58). Within the T2D population, reportedly 58–86% of individuals also present with OSA (70–72). Moreover, in individuals with existing T2D, a dose-dependent relationship is found between worsening glycemic control and the severity of OSA independent of obesity (68, 73, 74). If autonomic neuropathy is present alongside T2D, the individual is at an increased risk for mixed apneic events due to the degradation of respiratory neurons resulting in overall decreases in chemoreception and increased HCVR (75). While these statistics may suggest that T2D precedes the development of OSA, this hypothesis has not been supported by clinical longitudinal studies (142) or meta-analysis (143). Instead, the clinical data point to the likelihood that OSA exacerbates existing T2D through an insulin-related mechanism.

Within non-diabetic and T2D individuals, insulin resistance appears to be more closely tied with OSA than fasting hyperglycemia or glucose variability. Clinical association studies have generally found that insulin resistance is independently associated with OSA (52, 59–64), however data undermining this association, particularly from early clinical studies are present (144, 145). Much of the research exploring the relationship between insulin and OSA has been pioneered in rodent models utilizing IH. Indeed, chronic IH exposure leads to insulin resistance in lean rodents and exacerbates insulin resistance in diet-induced obese models (39, 77, 80, 90). IH can also affect β cell function, leading to augmented basal secretion and reduced glucose-stimulated insulin secretion (80, 146). Much of insulin resistance induced by IH has been attributed to elevated sympathetic activity (147–149), as pharmacological or surgical methods used to block the sympathetic response prevent the development of IH-induced insulin resistance (136, 150). Indeed, individuals with OSA demonstrate increased sympathetic nerve activity (151). Additionally, IH is observed to increase pancreatic oxidative stress and reduce β3-adrenergic receptor mediated insulin secretion (152). A possible mediator between elevated sympathetic activity and insulin resistance following IH may be increased lipolysis. Increased sympathetic outflow contributes to lipolysis, which in turn leads to elevated free-fatty acids and finally insulin resistance (153). This hypothesis is supported by data from animal models where pharmacological inhibition of lipolysis prevents IH-induced decreases in insulin sensitivity (81). A recent clinical study further demonstrated that lipoprotein abnormalities observed in OSA individuals are more directly related to insulin resistance than OSA severity itself (154). Upstream of lipolysis, hypoxia-inducible factor-mediated transcription (e.g., HIF-1α, HIF-2α) may play an important role in linking oxygen desaturation induced by IH with lipolysis (155–157) and/or insulin resistance (158).

While mounting evidence supports the conclusion that IH leads to insulin resistance, research on how decreased insulin sensitivity may lead to the development of OSA is scant due in part to the challenging experimental designs. One such way to specifically manipulate insulin is with the drug streptozotocin (STZ). STZ leads to apoptosis of pancreatic beta cells and, when given in low to moderate doses, is used as a model of T2D, reflecting insufficient insulin action and hyperglycemia. Interestingly, STZ-induced T2D (e.g., STZ-T2D) rats have marked reductions in ventilatory control, including reductions in the HCVR and the HVR, as well as increased incidents of apnea (96, 97). Insulin or metformin treatment can substantially improve disordered breathing in STZ-T2D rats (96, 97), suggesting that insufficient insulin action may contribute to the development of sleep apnea. However, it is possible that observed changes in chemoreception and disordered breathing are secondary to STZ-induced decreases in peripheral sympathetic activity (159) as opposed to insulin action *per se*. Along these lines, STZ-T2D rats exposed to chronic IH exhibit an attenuation in fasting hyperglycemia and mitigated (160) or improved (161) insulin resistance, perhaps reflecting the inability of IH to stimulate a sympathetic system dampened by STZ treatment. Notably, this effect in STZ-T2D rodents is distinct from IH's effect in diet-induced obese T2D animals, which experience an exacerbation in insulin resistance (39, 77, 80, 90).

If insulin action were central to the pathogenesis of sleep apnea, one might expect insulin deficient, Type 1 Diabetic (T1D) individuals to have a higher incident of disordered breathing. In support of this conclusion, children with T1D exhibit more total apneic events and increased CSA, associated with hyperglycemia and autonomic dysfunction (98, 99). Conversely, individuals with T1D are also at risk for a rare syndrome presenting with disordered breathing and hypoglycemia. Dead-in-bed syndrome is believed to occur due to initial bouts of nocturnal hypoglycemia associated with excessive hypotonia of the airway followed by IH, breathing depression, and finally cardiac arrhythmia (162). While these two conditions are distinct in insulin action, they share a common result on sympathetic function. Indeed, chemoreceptors at the carotid bodies are known to respond to elevated insulin with sympathetic activation (163, 164) while hyperglycemic events also cause autonomic dysfunction (99). These data suggest that it may not be insulin action *per se* associated with disordered breathing, but insulin's effect on the autonomic system. Beyond T1D and T2D individuals, other disease states associated with insulin resistance have increased risk of exhibiting disordered breathing. Women with polycystic ovary syndrome (PCOS) exhibit insulin resistance and are 30 times more likely to exhibit OSA compared to women without PCOS (100). Moreover, the insulin resistance displayed by PCOS individuals predicts OSA independent of obesity (101, 102). Hyperinsulinemia and hypoglycemia is also present in individuals with congenital central hypoventilation syndrome (CCHS), a syndrome associated with impairments in chemosensitivity and sleep disordered breathing due to a mutation in the *PHOX2B* gene (134). Individuals with CCHS also exhibit dysregulation to their autonomic nervous system which likely contributes to both their metabolic and disordered breathing phenotype (134). Taken together, these clinical studies suggest that insulin resistance may be an important contributing factor in OSA pathogenesis. However, it is difficult to determine the isolated role of insulin action as alterations in autonomic nervous system activity and/or chemosensitivity are often occurring simultaneously.

In most randomized clinical trials, CPAP treatment improves short-term insulin resistance (165), however the impact of CPAP on long-term insulin resistance is unknown (77). Long-term improvements in insulin action due to CPAP would support the hypothesis that OSA leads to or exacerbates insulin resistance and undermine the hypothesis that insulin resistance itself was leading to OSA. Echoing the latter, a recent randomized, placebo-controlled pilot study reported that manipulating insulin sensitivity via treatment with pioglitazone did not affect OSA (145). However, data from rodent models complicate these findings. In non-obese, high-fat diet fed rats, metformin treatment increased insulin sensitivity and prevented the development of sleep apnea independently of body weight (103). This discrepancy may be due to the specific type of apnea being studied. In rats, central apneic events occurring with relatively higher frequency than in the general human population. If this is true, further research into differentiating between obstructive, central, and mixed apneic events may yield differential contributions of insulin resistance.

Overall, ample evidence demonstrates that insulin resistance is associated with OSA independent of obesity, and that the cyclic bouts of hypoxia experienced by OSA individuals may be key to exacerbating insulin resistance. However, evidence demonstrating that insulin action alone leads to or exacerbates OSA is limited. One possibility is that insulin resistance is one of many factors affecting sleep disordered breathing and requires coincident impairments in the autonomic nervous system, glycemic control, or others (see leptin in the following section) to generate the conditions necessary for promoting OSA.

## Leptin

Leptin is a satiety hormone released by and in proportion to adipose tissue stores. The robustly positive relationship between leptin and body fat makes leptin an obvious confound when speculating on the root cause of OSA. In general, as leptin increases with fat mass, it acts as an anti-obesity hormone. However, too much leptin can lead to leptin resistance wherein the anti-obesity properties are no longer triggered. Indeed, treating obese individuals with peripheral leptin fails to reduce body weight (166). However, leptin resistance may not impact all of leptin actions. For example, even in obese individuals, leptin's action on sympathoexcitatory actions is maintained (167). It is possible that elevated leptin and/or leptin resistance observed in obesity may be contributing to OSA.

In non-T2D individuals, clinical studies have identified a positive association between OSA and leptin independent of body fat (61, 65). Though a causal relationship has not been defined, there is also evidence that both leptin resistance (168, 169) and OSA increase with aging (144). Healthy pre-menopausal women have significantly higher circulating leptin levels compared to men independent of body weight (168) and are also significantly less affected by OSA (0.6% of pre-menopausal females vs. 3.9% of males) (170), suggesting that increased leptin signaling or elevated leptin may be protective of OSA. However, this effect appears to be absent in post-menopausal women (171). Based on these association studies like these, if leptin action is involved in OSA, then the involvement of other endocrine systems including sex-hormones and insulin resistance may be important co-contributors to OSA.

Recently, accumulating evidence points to leptin action upstream of disordered breathing. Clinical data from individuals with obesity hypoventilation syndrome suggest that leptin resistance contributes to a reduction in HCVR and HVR likely via an impaired chemosensitivity (172). Leptin deficient *ob/ob* mice exhibit a disordered breathing phenotype (104), including a reduction in HCVR (105), and treating *ob/ob* mice with leptin improves ventilation within 3 days, before significant weight loss occurs (105). The obese Zucker rat, which lacks leptin receptors, also exhibit a decreased HVR (106) however maintain a stable upper airway during sleep (173). Leptin resistant New Zealand Obese mice exhibit inspiratory flow limitation, suggestive of sleep disordered breathing (107). These rodent data are partially recapitulated in individuals with lipodystrophy which exhibit chronically low levels of leptin (109, 110) and are at a greater risk to the development of OSA (111), suggesting that insufficient leptin action may lead to OSA in humans. However, lipodystrophic individuals also have increased fat deposits around the neck and exhibit characteristic insulin resistance (112, 113), making it difficult to determine the individual contribution of leptin on apneic events independent of physical body weight or other physiological variables such as insulin.

Leptin action may also be instrumental in downstream signaling of OSA. IH has been shown to lead to a significant increase in leptin levels in both rodents and humans (37, 85–89). Similar increases in leptin are observed in OSA patients (66) and in those with shortened sleep (76). CPAP treatment in OSA individuals tend to decrease leptin levels independent of body weight, however this is not consistently observed in all studies (128). As many patients lose weight with CPAP, noting changes in body fat specifically (174), is particularly important to consider when reflecting on leptin action. When exposed to IH, rodents with deficient leptin signaling have exacerbated insulin resistance (175) and increased cardiovascular impairments including endothelial dysfunction (176). Leptin treatment prior to IH reduces insulin resistance and hyperlipidemia and improves endothelial relaxation and vascular stiffness in *ob/ob* mice (175, 177). Most intriguingly, leptin treatment can mitigate IH-induced hyperlipidemia and cardiovascular outcomes in lean, wild type (177) suggesting that a boost in leptin signaling may prevent downstream cardiometabolic consequences of IH. As these studies focus on peripheral leptin treatment, it is unclear if leptin is acting primarily on peripheral or central targets. However, recent evidence that manipulation of specific neuronal leptin receptors can lead to tachypnea and a decreased HCVR (108) supports the hypothesis that neuronal leptin signaling may contribute to disordered breathing.

Given the role of leptin in ventilatory drive and the increases observed following IH, leptin may be acting by way of a counterregulatory mechanism in an attempt to improve disordered breathing. Some have proposed leptin is directly controlled by hypoxia (86). However, leptin's tight relationship with other key players in OSA, including obesity and insulin sensitivity (178), especially in T2D individuals (179), make it difficult to draw specific conclusions about the role of leptin in OSA. Key areas of leptin's involvement in OSA require further exploration, including leptin's action in chemosensitive regions, and the synergistic role of leptin, insulin, and other hormones on downstream cardiometabolic outcomes associated with OSA.

## Conclusions and future directions

The strong association between obesity, OSA, and T2D has led many to speculate about the bidirectional relationship between metabolic disease and OSA. A wealth of clinical studies suggests that OSA can exacerbate T2D, and animal studies have echoed this conclusion demonstrating that rodents exposed to IH show impairments in glycemic control, insulin resistance, and altered leptin levels. With the aid of these animal models, a number of mechanistic hypotheses have been posed which link OSA to the metabolic syndrome, including an elevation in sympathetic tone, increased lipolysis, inflammation (180), and reductions in chemosensitivity. A more debated hypothesis positions the physiological components of obesity, including glucose, insulin, and leptin signaling as key contributors to the etiology of OSA. While it is becoming clear that elements beyond the physical weight of body fat may be leading to OSA, the field is largely undecided on which factor(s) are critical to OSA's etiology. Novel hypotheses on this aspect of the directional relationship would do well to consider the synergistic relationship between insulin and leptin at the foundation for healthy and disordered breathing. Other new avenues of research show great promise in increasing our understanding of OSA and the relationship to cardiometabolic diseases. Emerging evidence that the gut microbiota is altered following IH (181), for example, elucidates a novel, potential link between OSA, glucose metabolism, and the gut (182). The involvement of the circadian biology with OSA and sleep disordered breathing also shows great promise (183). OSA individuals exhibit a circadian dysregulation of cortisol (184), and treatment with melatonin has been found to mitigate IH-induced hyperglycemia (185), insulin resistance, and microvascular damage (186). Research in these fields are on-going and may revel exciting new information about OSA etiology.

Advancing our knowledge on the etiology of OSA may lead to novel treatment strategies. Currently CPAP is the most effective and widely used treatment for individuals with OSA (27). Despite its low compliance (29), CPAP treatment modestly improves blood pressure (151), attenuates heart failure (187), and improves cardiac function (188, 189) and can significantly reduce mortality due to cardiovascular diseases (190). CPAP can also improve AHI (191) and blood oxygenation in individuals presenting predominantly with CSA (192). CPAP is unique in that it not only targets physical obstructions but also alleviates a brain-central failure to breathe. Indeed, the success of CPAP reflects the heterogenous nature of sleep apnea with both anatomical and neuronal underpinnings. For comparison, surgical treatments such as the Uvulopalatopharyngoplasty (UPPP) target anatomical obstructions and success rates are heavily dependent on degree of anatomical obstruction (193). Whereas drugs targeting the brain improve OSA (194), but not to the extent as CPAP. For example, fluoxetine (Prozac), a selective serotonin reuptake inhibitor commonly used to treat depression, in combination with ondansetron, improves apneic events by ~40% (195). Similarly, acetazolamide, a carbonic anhydrase inhibitor used to treat glaucoma and other conditions, has been shown to improve central sleep apnea and oxygen saturation (196). Taken together, the current treatment data supports a growing hypothesis that OSA involves more than physical anatomical obstructions and implicates a physiological component in the development of apneic events. Especially in the cases of mixed apneic events, more common in those with T2D (26), it becomes critical to understand the etiology of sleep apnea in order to effectively treat it beyond physical and anatomical obstructions.

## Author contributions

All authors listed have made a substantial, direct and intellectual contribution to the work, and approved it for publication.

### Conflict of interest statement

The authors declare that the research was conducted in the absence of any commercial or financial relationships that could be construed as a potential conflict of interest. The reviewer RA and handling Editor declared their shared affiliation.
